# ^1^H, ^13^C and ^15^N resonance assignments for the response regulator CheY_3_ from *Rhodobacter sphaeroides*

**DOI:** 10.1007/s12104-016-9703-x

**Published:** 2016-07-29

**Authors:** Lorena Varela, Christian H. Bell, Judith P. Armitage, Christina Redfield

**Affiliations:** Department of Biochemistry, University of Oxford, South Parks Road, Oxford, OX1 3QU UK

**Keywords:** CheY_3_, Response regulator, Chemotaxis, *Rhodobacter sphaeroides*, NMR resonance assignments

## Abstract

*Rhodobacter sphaeroides* has emerged as a model system for studies of the complex chemotaxis pathways that are a hallmark of many non-enteric bacteria. The genome of *R. sphaeroides* encodes two sets of flagellar genes, *fla1* and *fla2,* that are controlled by three different operons. Each operon encodes homologues of most of the proteins required for the well-studied *E. coli* chemotaxis pathway. *R. sphaeroides* has six homologues of the response regulator CheY that are localized to and are regulated by different clusters of chemosensory proteins in the cell and have different effects on chemotaxis. CheY_6_ is the major CheY stopping the *fla1* flagellar motor and associated with a cytoplasmically localised chemosensory pathway. CheY_3_ and CheY_4_ are associated with a membrane localised polar chemosensory cluster, and can bind to but not stop the motor. CheY_6_ and either CheY_3_ or CheY_4_ are required for chemotaxis. We are using NMR spectroscopy to characterise and compare the structure and dynamics of CheY_3_ and CheY_6_ in solution. We are interested in defining the conformational changes that occur upon activation of these two proteins and to identify differences in their properties that can explain the different functions they play in chemotaxis in *R. sphaeroides*. Here we present the ^1^H, ^13^C and ^15^N assignments for CheY_3_ in its active, inactive and Mg^2+^-free apo form. These assignments provide the starting point for detailed investigations of the structure and function of CheY_3_.

## Biological context

The process by which bacteria bias their motility, enabling them to move towards favourable chemical stimuli, such as nutrients, and away from unfavourable ones, such as toxins, is known as chemotaxis (Wadhams and Armitage 2004). Bacteria sense changes in the levels of these chemo-effectors and a signal is sent, via a two-component signalling pathway, to the flagellar motor to bias its direction of rotation.

Important insights into bacterial chemotaxis have been derived from extensive studies of the ‘relatively’ simple, single chemotaxis signalling network of *E. coli* (Baker et al. 2006; Eisenbach 2007; Hazelbauer et al. 2008). This pathway depends on autophosphorylation of a histidine protein kinase (HPK) in response to a signal from a sensor domain, with subsequent transfer of the phosphoryl group to the aspartate on response regulator (RR) proteins that bind to the flagellar motor and alter its direction of rotation. Specifically, transmembrane chemoreceptors, arranged in a large protein cluster close to the cell’s pole, signal changes in the extracellular environment to the cytoplasmically associated HPK, CheA, with CheW acting as a linker protein. A reduction in attractant activates CheA, and phosphoryl groups are transferred to two different RR proteins, CheY and CheB. CheY is a 14 kDa single domain RR that is conserved across motile species. It is formed by 5 α-helices and 5 β-strands surrounding a conserved phosphoryl accepting aspartate residue, and once phosphorylated leaves CheA and diffuses to the flagellar motor, binding to the FliM component of the motor to cause switching of rotational direction and, hence, a change from smooth-swimming to tumbling that enables the bacterium to reorient and bias swimming away from unfavourable conditions.

Most bacteria have significantly more complex chemosensory systems than that described above for *E. coli* and these are not currently fully understood (Geer et al. 2002; Pruitt et al. 2007). Although these systems use components that are broadly similar to those used by *E. coli*, there are often extensive modifications to the way the protein components assemble to form the chemosensory pathway. It is becoming increasingly apparent that chemotaxis does not function in isolation, but is an essential part of more complex sensory systems in which a range of environmental signals are sensed, and balanced responses in the form of changes in gene expression and swimming direction are produced (Porter et al. 2008).

The photosynthetic bacterium *Rhodobacter sphaeroides* has multiple chemosensory pathways formed by homologues of the *E. coli* chemosensory proteins. *R. sphaeroides* has emerged as a model system for studies of the complex chemotaxis pathways that are a hallmark of non-enteric bacteria. However, there are still several outstanding unresolved questions about the specific interactions between the different proteins involved in the chemosensory network. These more complex chemosensory systems have novel features including: (1) the ability to sense a wider range of stimuli via two, or more, distinct signalling clusters and to integrate these signals with other sensory information such as the metabolic state of the cell, and (2) the ability to tune the chemotaxis system to suit the needs of the cell under the prevailing environmental conditions (Porter et al. 2008), for example, oxygen is an attractant for aerobic cells but a repellent for photo-heterotrophically grown *R. sphaeroides* cells.

The genome of *R. sphaeroides* encodes two sets of flagellar genes, *fla1* and *fla2* that are controlled by three different operons encoding chemotaxis proteins. Each operon encodes homologues of most of the proteins required for the *E. coli* chemotaxis pathway. *R. sphaeroides* has a total of 13 chemoreceptors and has 4 CheWs, 4 CheAs, 6 CheYs and 2 CheBs which contribute to its complex signal-transduction pathway (Mackenzie et al. 2001). The six homologues of the response regulator CheY are localized to and are regulated by different clusters of chemosensory proteins in the cell and, while all bind the motor switch, they have different effects on chemotaxis. CheY_6_ is the major CheY stopping the *fla1* flagellar motor (Porter et al. 2002; Shah et al. 2000; Ward et al. 1995). CheY_3_ and CheY_4_, which are associated with the polar cluster, can bind to but not stop the motor, but one or the other of these is required for chemotaxis (Martin et al. 2001a, b; Porter et al. 2002, 2006). CheY_1_, CheY_2_ and CheY_5_ are all required for control of the *fla2* flagellum (Hamblin et al. 1997; Martinez-del Campo et al. 2011).

CheY from *E. coli* undergoes a conformational change upon phosphorylation of D57 by CheA switching it from an inactive to an active conformation that is able to bind to FliM with ~20-fold higher affinity. On the basis of a number of X-ray crystallographic and NMR studies, Y106 and T87 in *E. coli* CheY have been identified as the key residues involved in this conformational change which has been called the Y–T switch. In the inactive state, Y106 is solvent exposed which sterically hinders FliM binding. Upon phosphorylation of D57, T87 forms a hydrogen bond to the phosphate group and Y106 rotates into a buried conformation making FliM binding more favourable (Stock et al. 2000).

Comparison of the sequences of the six CheY proteins from *R. sphaeroides* with *E. coli* CheY highlights some interesting differences. T87 is replaced by a serine in all CheY’s except CheY_1_. None of the *R. sphaeroides* CheY’s has a tyrosine at position 106; CheY_1/3/4/5_ have a tryptophan at this position, CheY_2_ has a phenylalanine and, interestingly, CheY_6_ has a valine instead of an aromatic residue. In addition, CheY_6_ has a 10-residue insertion following β-strand-5 (which contains Y106 in *E. coli*), which is not found in CheY_1–5_ or in *E. coli* CheY.

Recently we have embarked on an NMR spectroscopy study to characterise the structure and dynamics of CheY_3_ and CheY_6_ from *R. sphaeroides* in solution. We are interested in defining the conformational changes that occur upon activation of these two proteins and to identify differences in their properties that can explain the different functions they play in chemotaxis in *R. sphaeroides*. Resonance assignment is the first step in any detailed study of protein structure and dynamics. Here we present the ^1^H, ^13^C and ^15^N assignments for CheY_3_ in its active, inactive and Mg^2+^-free apo form.

## Methods and experiments

### Protein expression and purification

CheY_3_ was expressed in BL21(DE3) cells. The expression vector (pQE-80) contained an N-terminal His6 tag for purification; this tag (MRGSHHHHHHG) was not removed for the majority of triple-resonance experiments used here for resonance assignment. A TEV cleavage site was later introduced to allow the removal of the His6 tag by TEV protease; this resulted in a better-resolved aromatic ^1^H–^13^C HSQC spectrum which facilitated assignment of the aromatic resonances, allowed more detailed analysis of the affinity of CheY_3_ for Mg^2+^, and was used for assignment of apo-CheY_3_ (Mg^2+^-free state). In both constructs, the single cysteine present in the sequence, C16, was replaced with serine to avoid aggregation problems.

^15^N-single-labelled and ^15^N/^13^C-double-labelled CheY_3_ was produced by initially growing cells in LB medium to boost the rate of cell growth. 2 mL of starter culture, grown in LB at 37 °C for ~15 h, was added to 1 L of medium containing 100 g/mL ampicillin. Cells were grown at 37 °C to OD_600_ ~0.8 and then collected by centrifugation (~9000*g* at 4 °C), washed with M9 salts buffer and resuspended into M9 minimal medium (25 % of the original volume) containing 1 g/L ^15^NH_4_Cl and 4 g/L ^13^C_6_-glucose (or unlabelled glucose for ^15^N single-labelled protein expression). Cells were then incubated at 30 °C for an hour, to allow them to adapt to their new growth conditions. Expression was induced with isopropyl-β-d-thiogalactopyranoside (IPTG) at a final concentration of 1 mM. Cells were grown at 30 °C for at least 12 h and then they were spun down (~9000*g* at 4 °C) and resuspended into 35 mL of 50 mM TRIS buffer at pH 8.0 containing 150 mM NaCl, 6 μL/mL of a 2.5 mg/mL DNase stock solution, 1.2 mg/mL of hen egg white lysozyme and one protease inhibitor cocktail tablet. The solution was incubated at 4 °C for 30 min. The cells were then disrupted using a French pressure cell (1000 psi) and the cell lysate was spun down (25,000*g* at 4 °C). As CheY_3_ is expressed in both the soluble fraction and as inclusion bodies, both the supernatant and the pellet were processed. The supernatant was ultra-centrifuged (~256,000*g* at 4 °C) and the pellet discarded. The inclusion body pellet was solubilised into 40 mL of denaturing buffer (50 mM TRIS, 150 mM NaCl, 6 M GuHCl, pH 8.0) with 0.5 % v/v Triton X100, stirring at room temperature for 30 min. The solution was then ultra-centrifuged (~256,000*g* at 4 °C) and the pellet discarded. Both supernatants (soluble protein and inclusion bodies) were loaded onto Ni^2+^ Fast Flow Chelating Sepharose columns (Amersham Biosciences). The protein from inclusion bodies was refolded on the column during the washing steps by gradually reducing the GuHCl concentration in the washing buffer. Both fractions were then eluted with buffer containing 50 mM TRIS, 150 mM NaCl and 200 mM imidazole at pH 8.0 and finally mixed after checking their purity using SDS-PAGE. Purified protein was dialysed against water and then lyophilized.

Where appropriate, the His6 tag was cleaved by adding 0.2 mg/mL of TEV protease and 5 mM β-mercaptoethanol to the protein elution fraction followed by dialysis against 2L of TEV reaction buffer for 15 h at room temperature and in the dark. The protein solution was then loaded again onto a Ni^2+^ Fast Flow Chelating Sepharose column to separate the cleaved CheY_3_ from the uncleaved protein as well as the His-tagged TEV protease.

### NMR spectroscopy

^15^N or ^15^N/^13^C-double-labelled samples of CheY_3_ were used for resonance assignment using standard protocols (Redfield 2015). This was carried out under five different experimental conditions: (1) 2 mM CheY_3_ in 20 mM sodium acetate with 2 mM MgCl_2_, at pH 4.5 (low pH inactive state); (2) 2 mM CheY_3_ in 20 mM sodium acetate, 2 mM MgCl_2_ and 4.5 mM BeF_3_^−^ [a phosphorylation mimic (Cho et al. 2000; Yan et al. 1999)], at pH 4.5 (low pH active state); (3) 0.7 mM CheY_3_ in 7 mM sodium acetate, 15 mM MgCl_2_, at pH 7.3 (high pH inactive state); (4) 0.7 mM CheY_3_ in 7 mM sodium acetate, 15 mM MgCl_2_ and 2 mM BeF_3_^−^, at pH 7.3 (high pH active state); (5) 0.7 mM CheY_3_ in 50 mM TRIS at pH 7.3 (no MgCl_2_) (apo state). All samples contained 95 % H_2_O/5 % D_2_O (v/v). NMR experiments were carried out at 293 K using three different spectrometers: a Bruker Avance 500 MHz spectrometer equipped with a TCI CryoProbe, a 750 MHz spectrometer equipped with either a home-built console and triple-resonance probe or a Bruker Avance II console and TCI CryoProbe.

Resonance assignments for the five different CheY_3_ samples were obtained using three-dimensional NMR experiments including ^15^N-edited NOESY-HSQC, ^15^N-edited TOCSY-HSQC, HNCA, HN(CO)CA, CBCANH, CBCA(CO)NH, HNCO, HN(CA)CO, HBHA(CBCACO)NH, (H)C(CCO)NH, H(CCCO)NH and HCCH-TOCSY. Details of the specific experiments used for each of the sample conditions can be found in the BMRB deposition files. The apo-state of CheY_3_ at pH 7.3 showed significant line broadening of numerous peaks in the ^1^H–^15^N HSQC. A 3D HCA(CO)N experiment was found to be useful for confirming ^15^N assignments in this sample when correlations involving broad ^1^H^N^/^15^N in standard triple resonance spectra such as HNCA and CBCANH were very weak.

NMR data were processed using NMRPipe (Delaglio et al. 1995) and analysed using CcpNmr Analysis (Vranken et al. 2005). ^1^H chemical shifts were referenced using the H_2_O peak (4.8 ppm at 293 K), previously calibrated with DSS, and ^13^C and ^15^N were referenced indirectly.

## Extent of assignments and data deposition

**Fig. 1:**
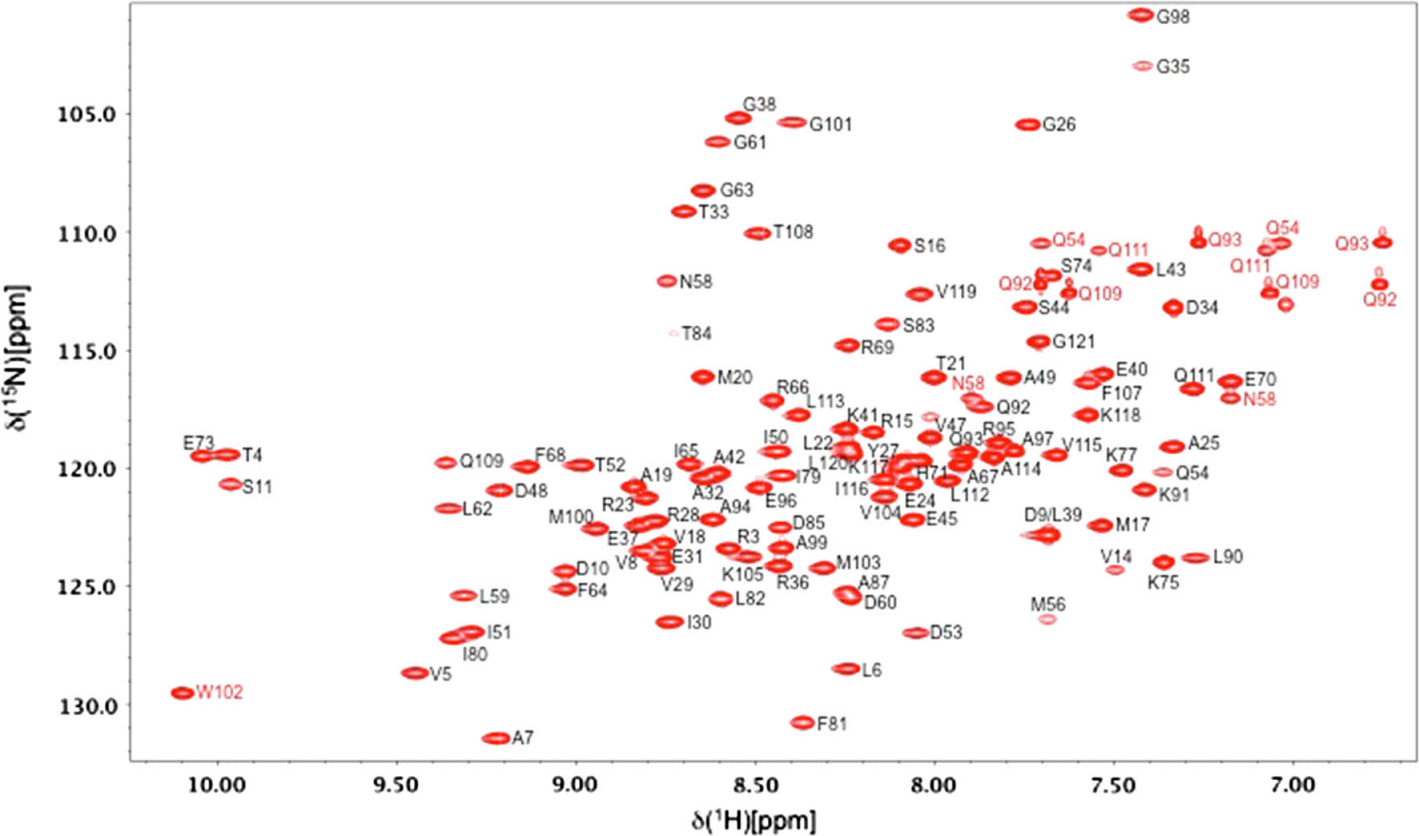
500 MHz ^1^H–^15^N HSQC spectrum of ^15^N–^13^C-labelled CheY_3_ (0.7 mM) in 7 mM sodium acetate (95 % H_2_O/5 % D_2_O), 15 mM MgCl_2_ at pH 7.3, 293 K. Peak assignments for backbone amides of residues 1–121 of the native sequence are indicated in *black*. Peak assignments for the side chains of N/Q/W are indicated in *red*

Figure 1 shows the ^1^H–^15^N HSQC spectrum of CheY_3_ in its inactive state at pH 7.3; assigned backbone ^1^H^N^/^15^N, asparagine/glutamine side chain ^1^Hδe/^15^Nδε and tryptophan indole ^1^Hε/^15^Nε are indicated. ^1^H^N^ and ^15^N backbone resonances for 105 of the 114 non-proline residues within the native sequence have been assigned for inactive CheY_3_ at pH 7.3. ^1^H^N^ and ^15^N assignments were not obtained for S1, S2, S13, N55, G76, S86, D88, T89 and W102. Upon addition of the phosphorylation mimic, BeF_3_^−^ (Cho et al. 2000; Yan et al. 1999), weak peaks corresponding to N55 and G76, and stronger peaks corresponding to S86 and W102 can be assigned; peaks for S1, S2, S13, D88 and T89 are still absent. Peaks for all backbone ^1^H^N^/^15^N have been assigned for both the inactive and active states of CheY_3_ at pH 4.5. The absence, or low intensity, of peaks corresponding to S1, S2, S13, N55, G76, D88 and T89 at pH 7.3 is likely due to their high intrinsic exchange rates (Bai et al. 1993) and the absence of hydrogen bonds to protect these amides. S86 and W102 may undergo a conformational change upon activation that leads to increased protection of their backbone amides. Extensive assignments have also been obtained for backbone (^13^Cα, ^1^Hα and ^13^C′) and side chain ^13^C/^1^H of both inactive and active CheY_3_ at low and high pH; generally the missing side chain assignments correspond to arginine, lysine, the ε-methyl groups of methionine and the aromatic ring of the four phenylalanine residues. The ^1^H–^15^N HSQC spectrum of the Mg^2+^-free apo state of CheY_3_ at pH 7.3 shows more extensive broadening than observed with Mg^2+^ bound; a lesser extent of assignment was achieved for this sample. The assignment statistics for CheY_3_ under the five different conditions used in the NMR studies are summarised in Table 1.

**Table 1:** Extent of assignment for CheY_3_ under various sample conditions

Sample conditions	Percent assigned					
	^1^H^N^/^15^N^a^	^13^C′	^1^Hα/^13^Cα	^1^Hβ/^13^Cβ	^1^Hγ/^13^Cγ^b^	^1^H/^13^C/^15^N^c^ (δ, ε, ζ, η)
CheY_3_ (inactive) pH 4.5, Mg^2+^	100/100	99.2	99.2/100	99.5/100	92.7/91.1	80.5/67.9/100
CheY_3_ (active) pH 4.5, BeF_3_, Mg^2+^	100/100	100	100/100	100/100	92.7/96.7	85.2/85.7/100
CheY_3_ (inactive) pH 7.28, Mg^2+^	92.1/92.1	96.7	97.7/98.3	96.8/97.3	89.1/90.0	79.7/76.2/87.5
CheY_3_ (active) pH 7.25, BeF_3_, Mg^2+^	95.6/95.6	97.5	99.2/99.2	98.9/99.1	88.3/90.0	82.0/82.1/100
CheY_3_ (inactive) pH 7.3, apo (no Mg^2+^)	84.2/86.8	83.5	93.8/95.9	89.4/92.0	71.5/76.7	37.5/38.1/62.5

^a^Assignment statistics are for residues 1–121 of the native sequence. The nitrogens for the 7 proline residues are not included in the statistics

^b^Gamma carbons from Asp, Asn, His, Phe, Tyr and Trp, which do not have attached ^1^H and are generally not assigned, are not included in the statistics

^c^Only δ, ε, ζ, η carbons with attached ^1^H are included in the statistics. Side-chain ^15^N/^1^H^N^ from Lys, Arg and His are not included in the statistics

**Fig. 2:**
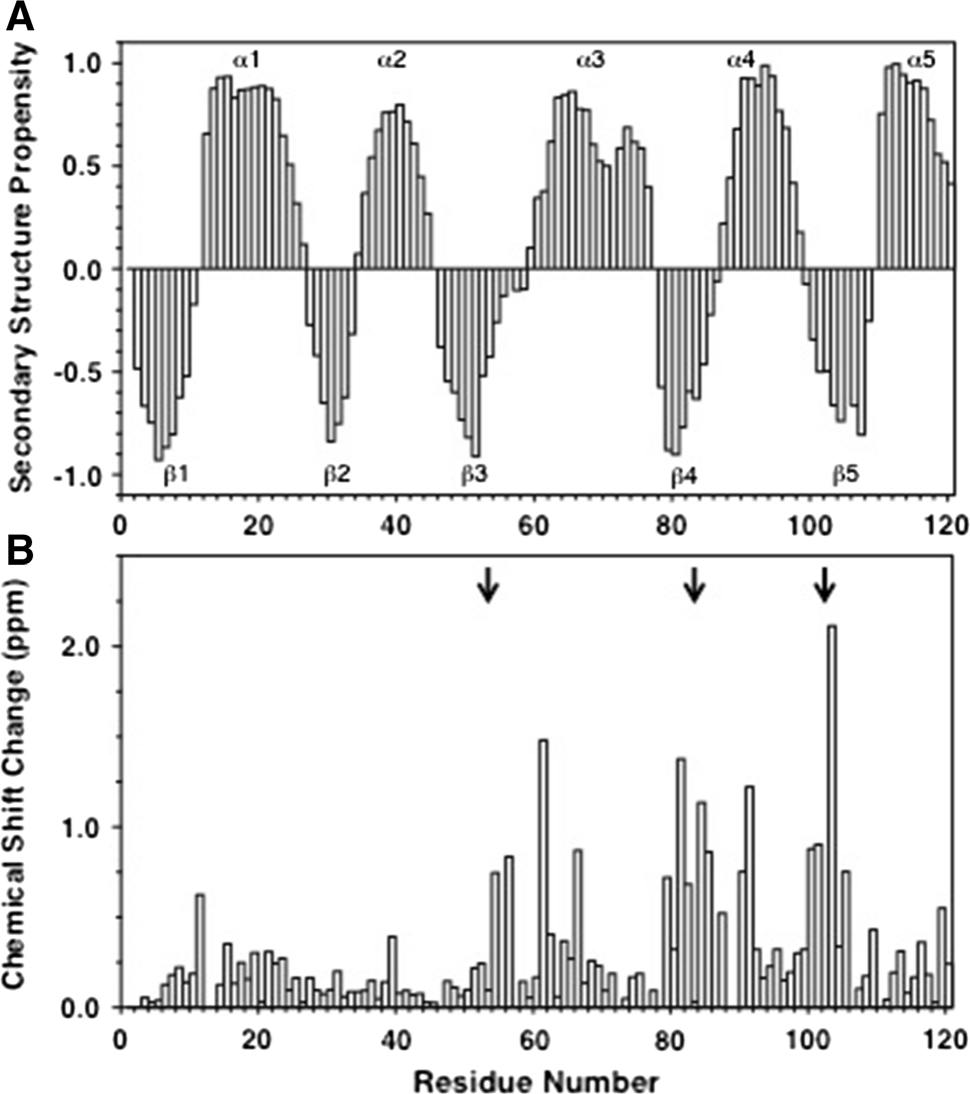
**a** Secondary structure propensities (SSPs), calculated from the ^13^Cα, ^13^Cβ, ^13^C′, ^1^Hα, ^1^H^N^ and ^15^N chemical shifts (Marsh et al. 2006), are plotted as a function of amino acid sequence for inactive CheY_3_ at pH 7.3. Positive and negative SSPs are indicative of α-helix and β-sheet structure, respectively. Similar analysis of the chemical shifts of CheY_3_ at pH 7.3 with BeF_3_^−^ indicates no significant change in secondary structure upon activation. **b** Combined chemical shift changes (Δδ_comb_ = [Δδ_HN_^2^ + 0.1[Δδ_15N_]^2^]^1/2^) observed for CheY_3_ at pH 7.3 in its inactive and active states (without and with the phosphorylation mimic BeF_3_^−^) are plotted as a function of amino acid sequence. The *black arrows* indicate residues D53, S83 and W102 which, by analogy with *E. coli* CheY, should be the binding site for BeF_3_^−^ (D57 in *E. coli*) and the pair of residues involved in the conformational switch (T87 and Y106 in *E. coli*)

The ^13^Cα, ^13^Cβ, ^13^C′, ^1^Hα, ^1^H^N^ and ^15^N chemical shifts have been used to predict secondary structure propensities (SSPs) for CheY_3_ in solution using the method of Marsh et al. (2006); these are plotted as a function of sequence for inactive CheY_3_ at pH 7.3 in Fig. 2a. The calculated SSPs show the expected alternating α/β pattern of secondary structure characteristic of the *E. coli* CheY fold. Phosphorylation of D53 of CheY_3_ by CheA_2_ leads to activation of CheY_3_ in vivo; this can be mimicked in vitro by adding BeF_3_^−^ (Cho et al. 2000; Yan et al. 1999). Significant changes in chemical shift are observed in the ^1^H–^15^N HSQC when BeF_3_^−^ is added to CheY_3_; the combined chemical shift difference (Δδ_comb_ = [Δδ_HN_^2^ + 0.1[Δδ_15N_]^2^]^1/2^) between the inactive and active states of CheY_3_ is plotted as a function of sequence in Fig. 2b. Significant chemical shift changes are seen in the vicinity of residues D53, S83 and W102 which by analogy with *E. coli* CheY should be the binding site for BeF_3_^−^ (D57 in *E. coli*) and the pair of residues involved in the conformational switch (T87 and Y106 in *E. coli*). Interestingly, more extensive chemical shift changes are observed for *R. sphaeroides* CheY_3_ than for *E. coli* CheY (Riepll et al. 2004) suggesting that the conformational switch upon activation in CheY_3_ may involve larger structural changes than in *E. coli* CheY. These assignments provide the starting point for detailed investigations of the structure, dynamics and function of CheY_3_.

The chemical shift assignments for CheY_3_ in its apo state (without Mg^2+^) at pH 7.3, in its inactive state (without BeF_3_) at pH 4.5 and pH 7.3, and in its active state (with BeF_3_^−^) at pH 4.5 and pH 7.3 have been deposited in the BioMagResBank (http://www.bmrb.wisc.edu) under the accession numbers 26789, 26778, 26769, 26777 and 26776, respectively.
